# An automated protocol for modelling peptide substrates to proteases

**DOI:** 10.1186/s12859-020-03931-6

**Published:** 2020-12-29

**Authors:** Rodrigo Ochoa, Mikhail Magnitov, Roman A. Laskowski, Pilar Cossio, Janet M. Thornton

**Affiliations:** 1 Biophysics of Tropical Diseases, Max Planck Tandem Group, University of Antioquia, 050010 Medellín, Colombia; 2 European Molecular Biology Laboratory, European Bioinformatics Institute (EMBL-EBI), Wellcome Trust Genome Campus, Hinxton, Cambridge CB10 1SD UK; 3 Department of Theoretical Biophysics, Max Planck Institute of Biophysics, 60438 Frankfurt am Main, Germany; 4 Present Address: Department of Biological and Medical Physics, Moscow Institute of Physics and Technology (National Research University), Dolgoprudny, Russia 141701

**Keywords:** Proteases, Peptides, Promiscuity, Bioinformatics, Structure

## Abstract

**Background:**

Proteases are key drivers in many biological processes, in part due to their specificity towards their substrates. However, depending on the family and molecular function, they can also display substrate promiscuity which can also be essential. Databases compiling specificity matrices derived from experimental assays have provided valuable insights into protease substrate recognition. Despite this, there are still gaps in our knowledge of the structural determinants. Here, we compile a set of protease crystal structures with bound peptide-like ligands to create a protocol for modelling substrates bound to protease structures, and for studying observables associated to the binding recognition.

**Results:**

As an application, we modelled a subset of protease–peptide complexes for which experimental cleavage data are available to compare with informational entropies obtained from protease–specificity matrices. The modelled complexes were subjected to conformational sampling using the Backrub method in Rosetta, and multiple observables from the simulations were calculated and compared per peptide position. We found that some of the calculated structural observables, such as the relative accessible surface area and the interaction energy, can help characterize a protease’s substrate recognition, giving insights for the potential prediction of novel substrates by combining additional approaches.

**Conclusion:**

Overall, our approach provides a repository of protease structures with annotated data, and an open source computational protocol to reproduce the modelling and dynamic analysis of the protease–peptide complexes.

## Background

Proteases are enzymes present in all species, from bacteria to vertebrates, and they account for approximately 2% of the genes in humans, second in number only to transcription factors [1]. These enzymes are involved in almost all fundamental processes in the cell, catalysing the cleavage of peptide bonds both in proteins and oligomeric peptides [2, 3]. Recognition and binding of a polypeptide substrate, which is cleaved at a specific peptide bond in the active site, occurs via pockets which accommodate specific amino acid side chains [3, 4]. The eight pockets, or subsites, are labelled S4-S1, S1ʹ-S4ʹ, with the corresponding peptide residues identified as P4-P1, P1ʹ-P4ʹ, the bond cleaved being the peptide bond between P1 and P1ʹ. The proteases can be grouped into serine, cysteine, threonine (covalent catalysis), aspartic and metallo-endopeptidases (general acid–base catalysis) according to the main types of reactive groups in the active site. The serine proteases are the largest and the best-studied class of proteases [5, 6]. They contain the classic Asp-His-Ser catalytic triad, displaying a generally accepted cleavage mechanism for a large number of diverse amino acidic substrates.

For some protease families, cleavage information can be obtained experimentally using methods such as mass spectrometry, liquid chromatography, N-terminal sequencing, and others [7]. Cleavage data provides clues about the enzyme’s promiscuity and specificity, while curated information can identify the sequence recognition patterns of the binding sites for known proteases [8]. Public databases, such as MEROPS, are repositories storing protease experimental data, including evidence of cleaved substrates, the biological context of their molecular reactions, ligands of pharmacological relevance and comparative genomics findings [9]. However, most of the knowledge is available only for a small set of well-studied families and subfamilies. This becomes a problem when studying under-represented protease classes participating in a biological process of interest for biomedical and industrial applications [10]. It would be valuable to predict peptide substrate motifs for any protease of a given sequence. Therefore, based on data for those proteases with identified cleavage patterns, computational prediction tools have been developed to identify potential substrates (as defined by a short peptide sequence) using as input any protease sequence [11].

Most of the available predictive methods rely on machine learning models trained with experimental cleavage data [12, 13], as well as bioinformatics pipelines able to quickly scan cleavage patterns in massive protein sequence databases [14–16]. However, these methods result in many false positives given the small set of proteases with representative data [17]. Despite these limitations, the methods are actively implemented by the computational biology community using public web repositories and tools [18–20]. To understand protease substrate specificity, researchers have focused on studying structural information available for multiple protease families [21, 22]. The serine and cysteine proteases are the most studied groups due to their roles as molecular machines involved in immune response, protein digestion, and signalling pathways, among other biological processes [23, 24]. Researchers have implemented structural studies to characterize small molecule inhibitors and drug-like entities such as modified peptide ligands, able to modulate these enzymes’ activity [25]. In the case of peptidomimetics, these have been designed to confer stability to peptide-based ligands and avoid cleavage of the molecule by the enzyme itself [26]. Because of this, many of the protease-peptide structural complexes that are available involve modified substrates and other types of ligands used for drug discovery purposes.

Hence, using structural data to identify drug-like molecules that might interact with given proteases still requires additional information [27, 28]. Researchers can derive models of protease-peptide complexes to analyse the binding spectrum [29, 30]. One approach is to analyse protease-peptide structures to ascertain amino acid preferences at different positions of a peptide substrate sequence [31]. These computational analyses usually employ experimental catalytic information stored in repositories such as M-CSA [32], bringing a clearer perspective of the structural role of the catalytic residues, and other pocket residues, during binding. A key factor is that the substrates are intrinsically flexible and can therefore change their conformation during binding, affecting the pose and the probability of cleavage [33]. To model this requires using simulations to help understand the protease/substrate’s dynamic behaviour.

The dynamics of molecular complexes have been studied using multiple approaches, including normal and enhanced molecular dynamics simulations [34, 35], as well as more computationally efficient alternatives such as Metropolis Monte Carlo, to simulate the dynamic behaviour of whole protein structures [36]. One example is the Backrub method of the Rosetta program, which uses restraints obtained from crystal structures available in the Protein Data Bank (PDB) [37]. The method allows the movement of certain bonds and angles after following an energy penalization criterion [38]. Such methods have been extensively used to study the effect of single point mutations for different protein systems [39], including proteases, and also to perform iterative mutation cycles on amino acids of peptide substrates to study the tolerance of these sequences to modification [40]. Such approaches have been used to predict the frequency and probability of finding amino acids at different positions of the peptide ligands, complementing experimental and sequence-based approaches to studying protease specificity [41].

To facilitate the structural and dynamical study of proteases bound to peptide substrates, it is crucial to have a set of annotated and curated structures, as well as protocols to model any peptide of interest through available templates. One problem is that it is difficult to obtain structures where the bound peptide spans the full length of the binding site, given the protease's natural impulse to cleave it. Hence a variety of means are used to prevent cleavage happening during co-crystallization, including use of non-natural amino acids or non-peptide residues. Nevertheless, it is common to find in the structural data bound ligands that only span the S4-S1 sites, with sites S1ʹ-S4ʹ empty—meaning there is a paucity of templates for helping model the P1ʹ-P4ʹ part of any peptide of interest. Here, we have annotated a set of protease crystal structures bound to peptide-like ligands to use as inputs to the structural analysis of their binding recognition. We have created a pipeline to model peptide substrates bound to protease structures using as templates co-crystallized peptide-like ligands. Any non-natural amino acids in the bound peptide are computationally replaced by natural amino acids occurring in known substrates, with any missing regions being reconstructed whenever possible. The fully annotated proteases, as well as the modelling/sampling code protocols, are provided for reproducibility purposes, and for running similar analyses with other proteases for which structures are available.

As an application of the method, we ran our pipeline on a set of protease structures, generating models of bound substrates, to identify any structural observables that might be a proxy for the reported experimental entropies. In each case, the modelled ligand was replaced by a family of potential peptide ligands, generated randomly. A simulation calculated binding energies and recorded various structural observables throughout the simulation. Finally, we compared the computational outputs with the experimental cleavage data (as defined by the specificity matrix) to explore which observables exhibit a similar trend. These could be used for further studies focused on predicting protease specificity profiles.

The main goal of this work is to provide an open and automatic protocol to model peptide substrates bound to different types of well-annotated proteases, facilitating the analysis of structural interaction observables.

## Results

### Annotation and modelling results

Based on the criteria used to annotate the proteins, we obtained a list of enzyme structures in complex to peptide-like ligands. The dataset was downloaded from the PDB and included 2798 unique structures. After annotating the dataset with additional information from other databases, 2581 entries were retained. From these, 758 were proteases (according to the EC classification) that had substrates in the catalytic site vicinity. Of this set, 599 structures belonged to one of the five main protease types included in the study (see “Methods” section). Furthermore, the metalloproteases had to be discarded because the energy functions and annotation pipelines are not configured for the presence of a catalytic metal in the binding site. Other proteases, with incomplete annotations, were also discarded, resulting in a final set of 310 structures. From this dataset, the serine proteases are the most representative group with the highest number of structures and a diverse set of subfamilies. A summary of the distribution is provided in Additional file 1: Fig. S1. Among the serine protease classes, thrombin (MEROPS id S01.217) and trypsin I (MEROPS id S01.151) are highlighted given the number of structures available and the experimental cleavage data reported in the database.

The full annotation of all the structures per protease family is available in Additional files 2, 3, 4, 5: Tables S1 to S4, and in the code repository. An example of the collected data, including the peptide amino acids, the pocket positions of each peptide amino acid, the numbering of the peptide residues in the PDB, the peptide chain and the MEROPS database id is provided in Table 1. In general, most of the peptides cover just one portion of the eight pockets, with lengths between 3 and 5 amino acids.

**Table 1:** Example of annotated structures including the PDB id, the amino acids, their positions in the pockets, the PDB numbering, PDB peptide chain and MEROPS id

PDB	Peptide amino acids	Pocket positions	PDB residue numbers	Chain id	MEROPS Id
1gdn	GAK	S3-S2-S1	1-2-3	B	S01.103
1hpg	AAPE	S4-S3-S2-S1	301-302-303-304	B	S01.267
1qix	VEPI	S4-S3-S2-S1	4-5-6-7	A	S01.153
1smf	CTKSI	S3-S2-S1-S1ʹ-S2ʹ	9-10-11--12-13	I	S01.151
2qa9	DAIY	S4-S3-S2-S1	1-2-3-4	I	S01.262
2wpm	EGR	S3-S2-S1	1-2-3	L	S01.214
2zgh	KVPL	S4-S3-S2-S1	4-5-6-7	B	S01.139
4boh	KPR	S3-S2-S1	52-53–54	M	S01.217

The subsequent modelling analysis focused on two types of proteases: serine proteases and cysteine proteases. Neither aspartic nor threonine proteases were included in the modelling due to some restrictions. For example, in the case of the aspartic proteases, only HIV protease structures are available in complex with multiple ligands, which include small molecules as inhibitors. For threonine proteases, most peptide-like substrates contain more than one modified amino acid, thus requiring the modelling of most of the substrate conformation. In addition, the serine and cysteine proteases have more structures and cleavage-pattern data available, which is helpful for assessing the impact of the structural observables on binding recognition. For the serine and cysteine proteases models, details of the template sequence, the sequence to model, and the substrate UniProtKB id are provided in Additional file 1: Tables S5 and S6 respectively. As a summary, the total numbers of serine and cysteine protease structure subsets that passed the filters, which were partially (only P4-P1) and fully modelled (P4-P4ʹ), are available in Table 2.

**Table 2:** Number of serine and cysteine protease structures remaining with peptides partially (P4-P1) and fully modelled (P4-P4ʹ)

Category	Serine proteases	Cysteine proteases
Total number of structures after the annotation	117	95
Structures remaining with partially modelled peptides (P4-P1)	69	64
Structures remaining with fully modelled peptides (P4-P4ʹ)	34	23

During the modelling process, most of the original crystal structures were discarded as the NNAAs bound could not be replaced by natural amino acid analogues. In addition, most of the protease-peptide crystal conformations are missing the P1ʹ-P4ʹ portion of the substrate. Because of this, some protein structures do not allow the correct modelling of the missing part, generating clashes with the protein amino acids located in that area. Those complexes were omitted too, leaving a final list of 34 serine proteases and 23 cysteine protease models, respectively.

### Selection of models for binding recognition analysis

To run the binding recognition analysis, we required proteases with a large amount of experimental cleavage data. Therefore, we focused this analysis only on a subset of serine proteases (MEROPS ids S01.217, S01.010, S01.151 and S01.131), which covers a diverse set of classes with available experimental data. This was not possible with the cysteine protease models, because all the structures are caspases with limited experimental information.

Regarding the serine proteases subset, the criterion for selecting the protease was prioritizing the one structure per class with most of the peptide amino acids present in the original crystal structure. Therefore, we included in the set the protease used as reference (PDB id: 3tjv), which is the only protease structure that is bound to a full 8-mer peptide substrate. The dynamic analysis was run using the selected models shown in Table 3.

**Table 3:** Number of serine protease structures that remained after the modelling of NNAAs and the missing flanking amino acids

PDB	MEROPS	Template	Model	Substrate id*	Set
1iau	S01.010	IEPD—	IEPDTDAP	P50502	Entropy
1ppg	S01.131	AAPA—	AAPAAAPP	P16403	Entropy
1tps	S01.151	-LTREL–	FLTRELAE	P23396	Entropy
4dt7	S01.217	VDPRL—	VDPRLIDG	P04070	Entropy
1ycp	S01.217	-GVRGP–	GGVRGPRV	P02671	Validation
2age	S01.151	AAPR—	AAPRERTT	Q13895	Validation
1ekb	S01.156	-DDK—	DDDKIVGG	P35030	Test
2zck	S01.162	-SQY—	SSQYSNTE	P04279	Test
3tjv	S01.147	PTSYAGDD	PTSYAGDD	Q1D7F8	Test

The MEROPS id, UniProtKB id and the peptide sequences after modelling are provided. In addition, the complexes are classified into three groups: (i) the structures with experimental data to calculate entropies, (ii) a validation set to calculate the descriptors with more than one structure per subfamily class and (iii) a test set to compare the trends of the selected structural observables with those calculated in the previous groups

^*^UniProtKB id

**Fig. 1:**
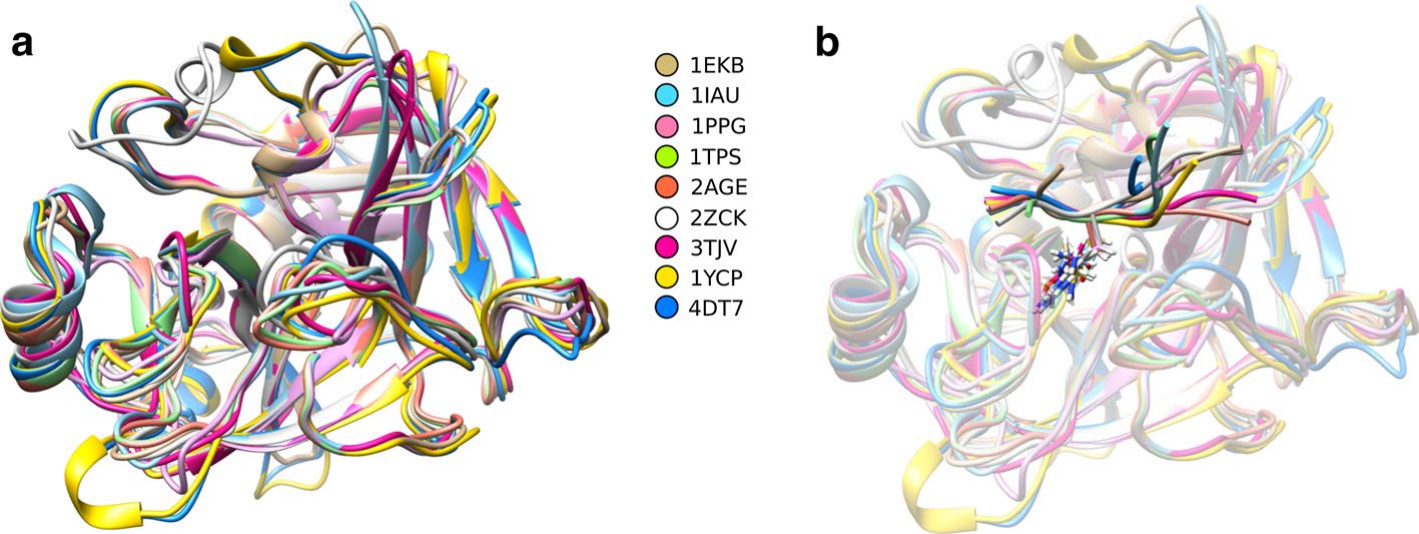
Structural superposition of the serine protease structures used in the analysis. **a** Alignment of the crystal structures without the bound peptides. **b** Superposition of the modelled peptides in cartoon format, with an atom representation of the peptide P1 position that interacts with the protein catalytic residues

To compare the conformations of the modelled peptides, a structural alignment was performed on the protease structures and the bound peptide conformations (Fig. 1). As expected, the structures share the same conformation with some variations in the loops, but the peptides tend to differ at positions P1ʹ to P4ʹ, mostly because of the lack of coordinate data for this substrate region in the original crystal structure dataset.

### Analysis of the observables and binding recognition insights

**Fig. 2:**
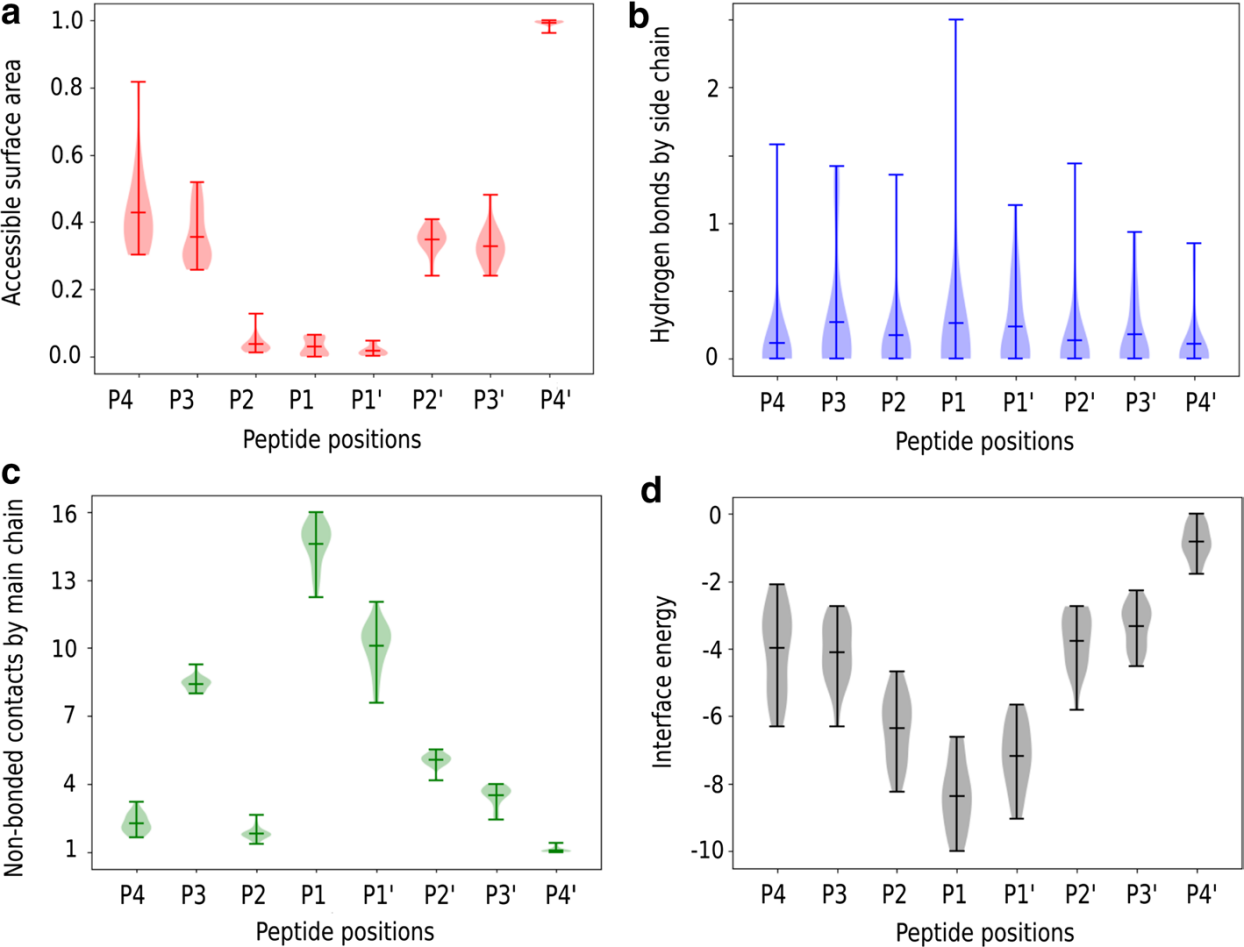
Distribution of the average observable at each position of the peptide substrates modelled in the structure 4dt7 (MEROPS id S01.217). The observables included are **a** the relative ASA, **b** the number of hydrogen bonds made by side chain atoms, **c** the number of non-bonded contacts made by main chain atoms and **d** the interaction energy calculated using the Rosetta scoring function

As a first step, the selected serine protease structures (PDB ids 4dt7, 1iau, 1tps and 1ppg) with experimental cleavage information available were subjected to refinement of the complex using the FlexPepDock protocol, and subsequent sampling with the Backrub approach from Rosetta. We calculated the observables for all the snapshots of the trajectory and visualized the normalized averages for all the amino acids at each position for every observable. Given that the initial peptide library was designed to contain an equal contribution of all the amino acids at all peptide positions, we focused on analysing general tendencies of the descriptors per peptide position. Longer and more exhaustive analyses are required to correlate in detail the frequency of each amino acid and the probabilities of being part of a substrate found in a protein, which is already explored by other methodologies such as the Sequence Tolerance protocols, among other alternatives [38, 40]. Here we focused on the normalized average values per peptide position, which is represented in the following example for the thrombin structure with PDB id 4dt7 (MEROPS id S01.217) in Fig. 2.

Here it is possible to notice differences in the variability and values assigned depending on the peptide position. For example, in the case of P1 and P1ʹ, positions interacting with the catalytic site, the amino acids tend to be completely buried, and the interactions are characterized by hydrogen bonds of the side chain, non-bonded contacts of the main chain, captured in the strong interaction energies. In the case of the flanking amino acids, the interactions are reduced, but their exposure to the solvent can be crucial in determining if the position can be highly promiscuous or not. This is the case at position P4, which has a reduced number of interactions and high accessibility to the solvent. In this case, it is possible to infer that the chemical nature of the side chain is not relevant for substrate recognition and any amino acid is tolerated at this position.

**Fig. 3:**
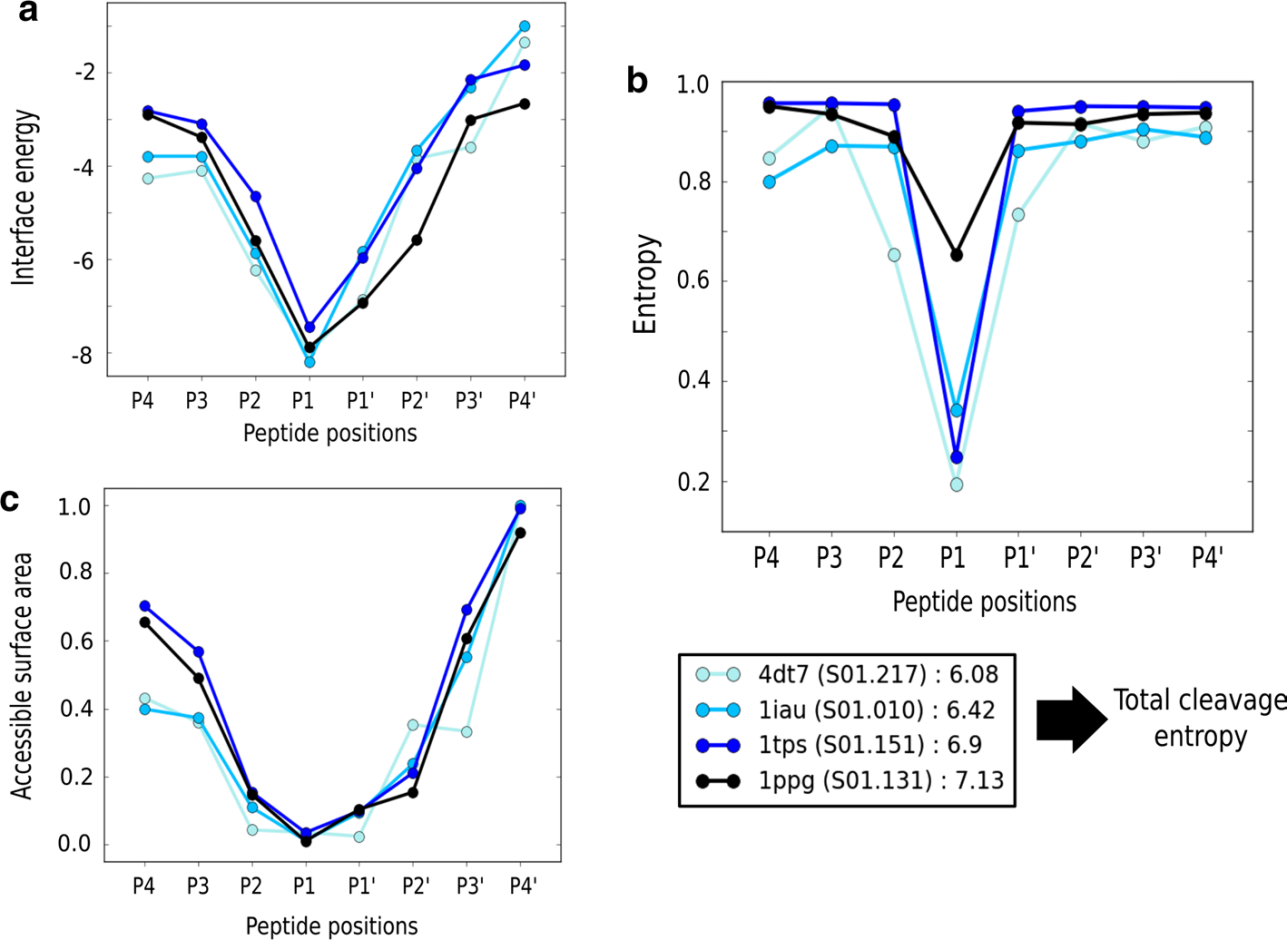
Average values of structural observables at each position of the peptides modelled for structures: 4dt7 (MEROPS id S01.217) in turquoise, 1iau (MEROPS id S01.010) in sky blue, 1tps (MEROPS id S01.151) in blue, and 1ppg (MEROPS id S01.131) in black. The observables included are **a** the interaction energy calculated using the Rosetta scoring function, **b** the relative accessible surface area, ASA, and **c** the entropy per position calculated from experimental cleavage data

After a general overview, we focused on the crystal structures having experimental cleavage data. Here, we analysed the observable averages per position, and compared the trends in the observables for all the structures with the information entropies calculated for each MEROPS subfamily/class based on the reported specificity matrix. The analysis was made using four serine proteases for which there is a massive amount of experimental cleavage data, providing a more robust measure of the entropy per substrate position. This gives a more sound comparison against the analysis of the structural observables. Among the calculated observables, the accessible surface area (ASA) and the single interface energy of each peptide position displayed trends that were the most similar to those described by the experimental entropies (Fig. 3). On the other hand, the contacts (H-bonds and non-bonded interactions) showed a more chaotic behaviour by peptide position, which reflects the modelling and sampling assumptions for the presented analysis. A visualization of the distribution of hydrogen bonds and non-bonded contacts are shown in Additional file 1: Fig. S2.

The trends suggest that the ASA and interface energy can be used to infer the influence of certain amino acids at the most promiscuous positions surrounding the region interacting with the cleavage site (i.e. P1 and P1ʹ). Both observables are reasonable options given the exposure of the protease pockets, and the fact that the interface energy covers not only contact contributions, but also a sum of empirical and physics-based terms that provides a better perspective of the binding process. The comparison was also analysed numerically after running the average Spearman correlation per position on the substrate for the selected structures (Additional file 5: Table S7). We found that the only observables with positive average correlations were the relative ASA (0.33) and the interface energy (0.22). However, it is not possible yet to correlate with accuracies higher than 0.5 the entropies associated to each pocket position. The latter might improve if more exhaustive sampling and energy calculations are used to discriminate between amino acids at each binding site pocket position. In addition, having more experimental data covering many different peptides would provide a better training and test set.

A similar analysis of the observable trends for the interaction energy and the relative ASA was run using all the models included in the study. A graphical representation of the values is provided in Additional file 1: Fig. S3. In all the cases, the observables show similar trends that can be extrapolated to identify which structural drivers can help to understand the binding recognition of substrates.

**Fig. 4:**
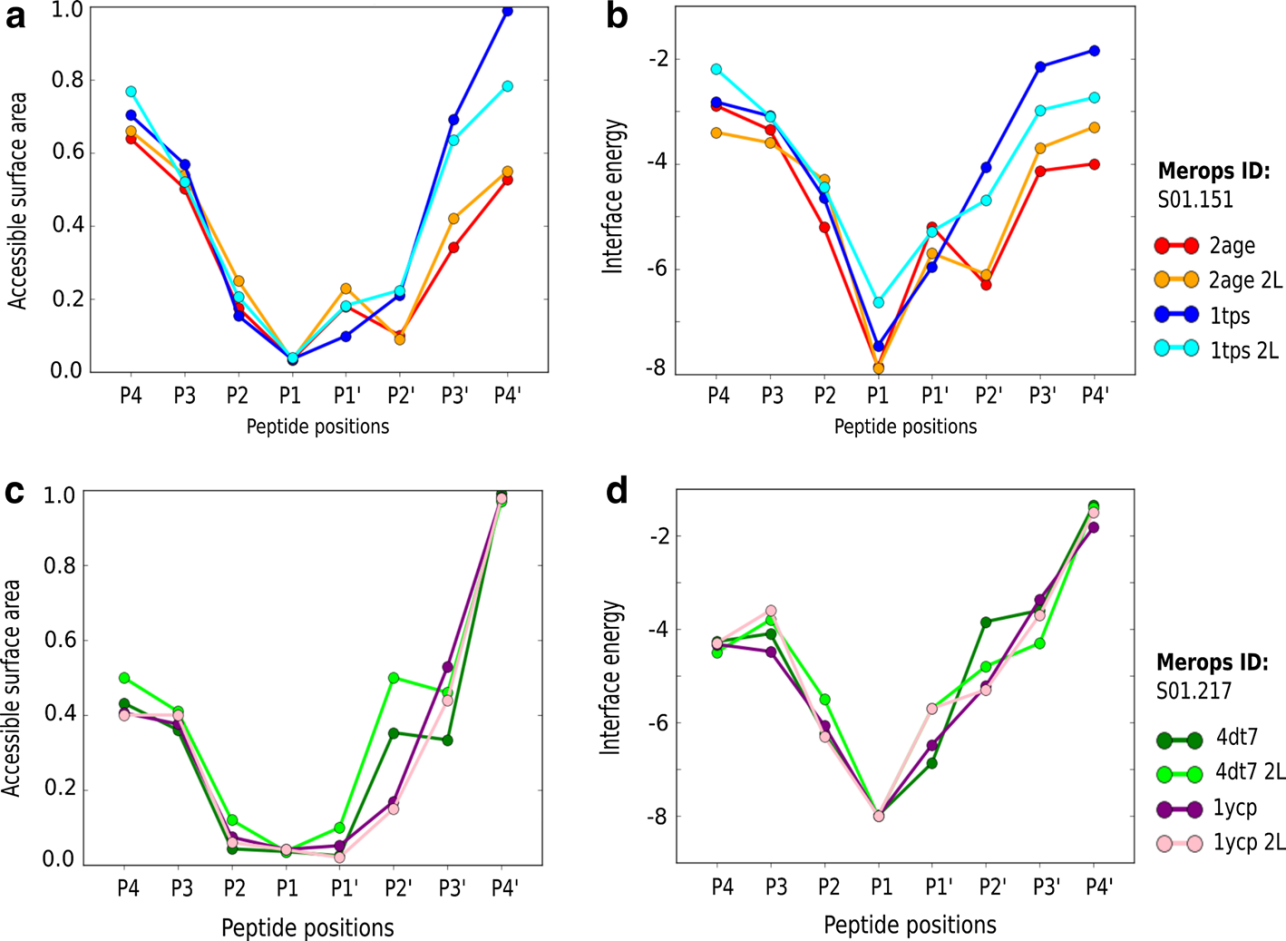
Average values of **a** the relative ASA at each position of the peptide substrates modelled for structures 2age (red and orange) and 1tps (blue and cyan) with MEROPS id S01.151 (trypsin I) from two independent random peptide libraries, and **b** the interaction energy calculated using the Rosetta scoring function. Similarly, the mean values of **c** the relative ASA at each position of the peptide substrates modelled for structures 4dt7 (green and lime) and 1ycp (purple and pink) with MEROPS id S01.217 (thrombin) from two independent random peptide libraries, and **d** the interaction energy calculated using the Rosetta scoring function. The second library is named as 2L in the figure labels

Finally, to support the usage of any structure per class to obtain similar results, we compared different structures belonging to the same subfamily class, as well as two independent random peptide libraries. This analysis allowed us to check if the protocol can be applied to any structure and model available in the PDB, and also to understand the independence of the results if different peptide library datasets are used to run the sampling protocol. The initial peptide-protease complex was refined to avoid bias of the initial structures. In Fig. 4 we can observe the trends covering two structures of the main two serine protease families: S01.217 (thrombin) and S01.151 (trypsin I). As expected, the plots are similar, with the cleavage region being less exposed to the solvent and with a higher interface energy associated. In some positions (i.e. P1ʹ or P2ʹ) the averages can vary a little from the expected behaviour, but the general consistency allows us to propose these metrics to study any other protease-peptide complex using the code protocols provided.

## Discussion

Here we provide an open source protocol to model peptide substrates bound to well-annotated structures of proteases, which can be applied to help gain an insight of their binding recognition using a structure and dynamic-based approach. We focused on a subfamily of serine proteases of biological importance, with emphasis on a subset for which experimental cleavage data are available. After modelling a random library of peptides based on reconstructed peptide substrates bound to protease structures, we found that structural observables such as the accessible surface area (ASA) and single-residue interface energies show a similar trend as the information-entropies derived from the protease specificity matrices. In the same way, we suggest that other peptide sequences can be studied from a structural perspective to determine which may be potential substrates of a protease of interest.

Modelling peptide substrates bound to proteases has been done before [29, 59, 60], but the methods have not been scaled up to run the sort of massive analysis we have performed in this work. The initial modelled conformations are subsequently improved by subjecting the models to dynamic analysis to re-accommodate potential wrong peptide conformations. The code provided is a meta-protocol that takes advantage of various open source projects, aiming to obtain reliable models with efficient computational times using local infrastructure. It facilitates the modelling of other complexes and allows the optimization of our proposal through, for example, the increase of sampling time or the addition of tools able to refine the models and in silico findings. The code is portable, and the dependencies can be easily installed to predict the models and run the structural analysis. The computational time for a single substrate/protease can be completed in a local machine/laptop in approximately 10 to 20 min. For large-scale analysis, using libraries of hundreds of peptides, it is recommended to use a high performance computing infrastructure, which can be optimized by sending jobs in parallel: one substrate per node. The main goal of the protocol is to contribute to the understanding of the enzymes’ specificity toward their substrates without exclusively requiring knowledge-based strategies, particularly for novel proteases without experimental data available.

Understanding the basis of substrate promiscuity of single proteins, such as proteases, and within whole families, is becoming important in protein design and directed evolution experiments, which can lead to new catalytic functions in old enzymes [61]. For example, some enzymes exhibit a high specificity towards their substrates, fine-tuned by evolution to allow them to distinguish between functional groups, isomers, and polymer units. Others, however, do not follow this classical paradigm and are able to catalyse distinctly different ligands [62]. It has been suggested that such substrate promiscuity could have an important role in enzyme evolution, where they exhibit different levels of specificity, ranging from completely specific to very broad [63]. One way to quantify this is through the analysis of experimental cleavage data, which can be limited depending on the protease of interest. Therefore, it is necessary to develop de novo computational approaches to infer the specificity of these enzymes [64]. In this work, we propose simple observables such as the relative ASA and the Rosetta calculated energy, which can be embedded in more elaborated pipelines able to discriminate with more certainty if novel sequences are susceptible of being cleaved by a reference protease.

In fact, our method can provide tools to study the specific frequency and probability of each amino acid at each position in a peptide substrate. However, to have a better performance, it will be required to combine multiple observables with other parameters, as well as model and sample libraries of known substrates instead of libraries of random peptides with equal contribution per amino acid per position [31]. Molecular mechanics can capture, in part, the essence of the interactions, but other considerations associated with the intrinsic catalytic reactions should be included to increase the accuracy of the calculations [65]. Published protocols such as the sequence tolerance methodology [40], and other energy-based approaches, can infer the amino acid contribution, but more exhaustive or hybrid methodologies are required to successfully predict novel substrates in comparison with the available sequence-based methodologies [66]. In addition, more thorough sampling, applied massively to many peptide binders, can positively influence the conformational landscape of the peptides, and capture additional information that can be better correlated with the experimental specificity data.

Finally, a crucial aspect to complement the binding studies is the availability of more structural complexes of proteases bound to peptide substrates. In this case, the straightforward approach is attempting to model known peptide substrates given the experimental information available. Here, we obtained a representative number of complexes that were modelled, but there is still a large set of protease structures that cannot be rigorously studied due to the presence of multiple non-natural amino acids and chemical modifications in the bound ligands. Because of this, it is important to parameterize as much as possible amino acid variants to allow the inclusion of modified chemical structures, improving the virtual screening of peptidomimetics and other types of modified substrates for drug discovery purposes [67]. The annotated set of structures, and the computational protocols provided, can help accelerate the task of predicting protease substrates and supporting the engineering of novel enzymes for medical and industrial applications.

## Conclusion

Based on the modelling and simulation approach proposed in this work, it is possible to model proteases in complex with peptide substrates and use the information to study their binding recognition. Calculated observables such as the relative ASA and the interface energy are suitable descriptors that can be compared with the experimental cleavage data of each pocket in the binding site. However, more exhaustive calculations are needed to discriminate specificity profiles between proteases by improving the correlations, and potentially use the information to predict the interaction with unknown substrates. In the case of researchers working on structure-based approaches with proteases, the descriptors can be useful also to filter peptides from large combinatorial libraries based on the propensity to generate certain types of interactions or being exposed to the solvent at different substrate positions. The code provided is user-friendly, open source, and can be implemented to study novel substrates and protease structures available in public repositories.

## Methods

The methods are split into two parts. The first describes the selection and annotation of protease crystal structures, and subsequent modelling of the peptide substrate binding region using the crystal structures as templates. The second uses the methodology described in the first section to model a set of proteases and compare various structural features with against the experimental cleavage data to see if any features might provide a predictor of strength of binding.

### Annotation and modelling of protease structures bound to peptide substrates

**Fig. 5:**
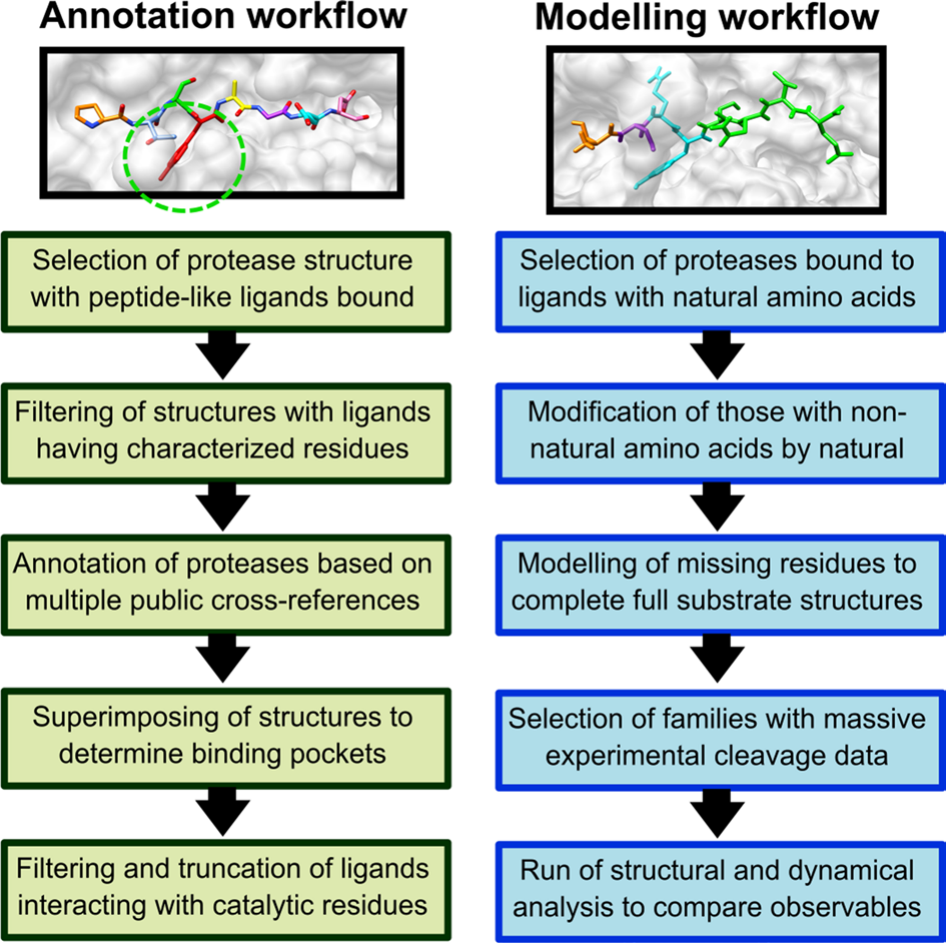
Summary of the main annotation and modelling workflow steps. The left-hand image represents a peptide-like ligand, bound to a protease, with the P1 position circled in green. The right-hand image shows a partial peptide bound to a protease where the missing residues (green) have been modelled to fill the full peptide binding site

This part of the protocol includes two main workflows: the first involves the annotation and filtering of protease structures, and the second uses the annotated proteases to model in various peptide substrates. A summary of the main workflow steps is shown in Fig. 5.

### Annotation workflow

#### Dataset of protease structures

Initially, a set of enzyme structures was downloaded from the Protein Data Bank (PDB) [37] based on their assigned Enzyme Commission (EC) numbers. The selected proteases were those that had been co-crystallized with ligands of between 3 and 12 residues, and where at least one residue was classified as peptide-like in the Chemical Component Dictionary [42]. The structures containing only unknown residues were removed from the initial list. The remaining entries in the dataset were annotated by identifying the protein chains directly interacting with the ligands, using information from PDBsum [43]. Structures that did not have this information were discarded. The protein chains identified were then annotated with their EC number, UniProtKB [44] and MEROPS ids [9], and Pfam [45] and CATH domains [46] of the regions interacting with the ligand. Where the data from the databases was misleading (e.g. same chain letter had multiple UniProtKB ids, or did not have any), the entry was dropped. To get a list of the catalytic residues and their roles, we used data from M-CSA [28] and PDBsum. For the chains that did not have catalytic residues reported in either database, we performed a homology search by creating pairwise sequence alignments with the annotated chains using ClustalW [47]. If an alignment resulted in more than 50% sequence identity, the catalytic residues and their roles were inferred from the target sequence to the query. For practical purposes, the first alignment satisfying the identity threshold was the one used.

#### Identification of substrates in catalytic sites

To check if the bound ligands were in the vicinity of the catalytic site, we used the NACCESS program (http://wolf.bms.umist.ac.uk/naccess/) to calculate solvent accessible areas (SAS) of protein chain residues with and without the bound ligand. If the accessibility of the catalytic residues changed after ligand removal, we considered the substrate to be in the catalytic site, and hence included the structure in our data set. The accessibility threshold used was 0. Structures lacking annotated catalytic residues were not analysed.

#### Annotation of protease amino acid binding pockets

**Fig. 6:**
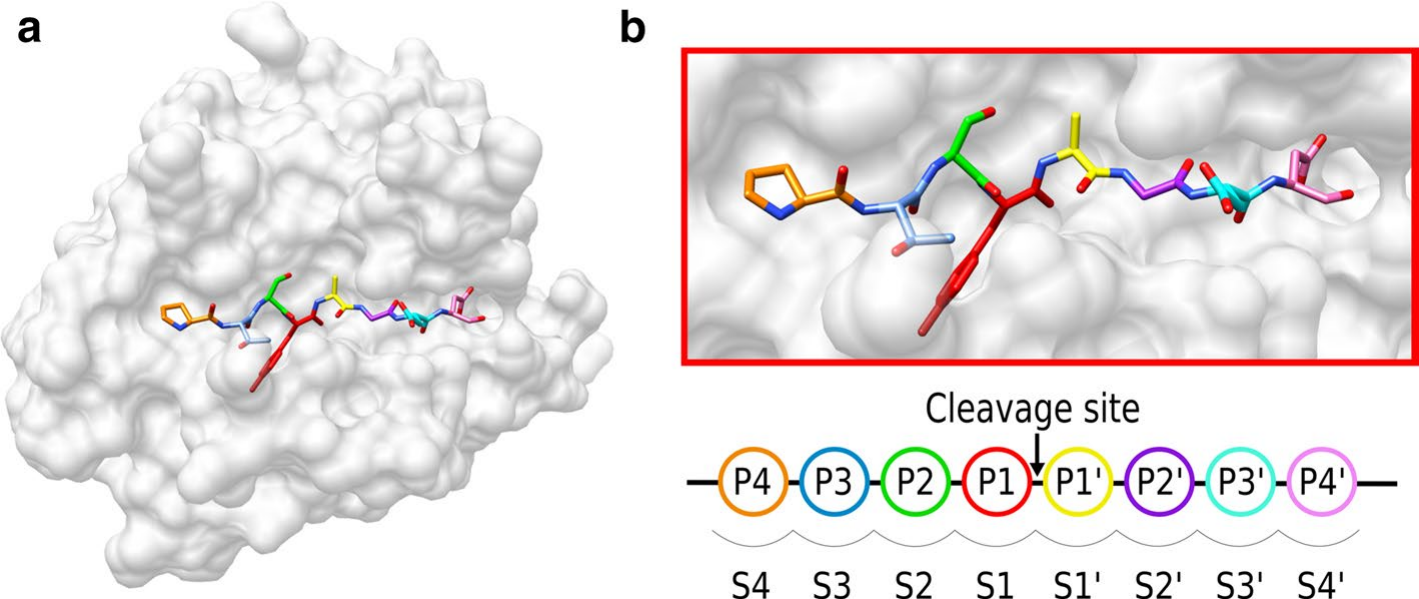
Schematic of a protease bound to a substrate, with the positions named according to the location of the cleavage site. **a** Peptide ligand bound to PDB entry 3tjv, a human serine protease granzyme H crystal structure. **b** ‘Zoomed in’ representation of the peptide coloured based on the positions interacting at the binding site

To identify the pocket residues where the substrates bind to the enzyme, we used the coordinates of the P4-P4ʹ residues of a reference substrate (PDB id: 3tjv) for the serine proteases (Fig. 6). This reference was chosen because the peptide substrate spans all the S4-S4ʹ pockets, and was used to annotate the protease pockets. The reference structure used for cysteine proteases was PDB id: 2j32; for threonine proteases was PDB id:4qby; and for aspartic proteases was PDB id: 4obf. For each reference peptide residue, we identified residues in the protease that had at least one atom within a 4.5 Å cut-off of the ligand. The basis of this threshold is the 5 Å maximum distance reported between a peptide and the enzyme’s catalytic residues for the nucleophilic attack [48]. These groups of protease residues were annotated as the S4-S4ʹ pockets according to the annotation of the closest reference residues (P4-P4ʹ). Some residues could belong to several pockets. We listed all identified pocket residues as forming the binding site of the reference protease. To annotate the pockets in other structures of the family, we performed structural alignments using PDBeFold (http://www.ebi.ac.uk/msdsrv/ssm/).

#### Final versions of the annotated structures

To annotate substrate residues with the pockets to which they bind, the reference substrate was projected to other structures using the generated structural alignments. The target residue with the most atoms around the reference residue within a 2 Å cut-off was assigned to the same pocket as the reference. The annotated complexes were subjected to the removal of acetyl or diazomethane groups, which are sometimes added to the substrates prior to crystallization. In some cases, gaps appeared between aligned target residues. For example, the first residue might be assigned to the S4 pocket, whereas other residues might be assigned to pockets S2 onwards—with no residue assigned to S3. In such cases, a manual re-annotation of the target pockets was done to close the gaps, by moving the shorter part of the substrate to join the longer part, which was taken to have been properly assigned. Finally, substrates that were partially aligned were truncated for further analysis. Substrates with fewer than three residues in the binding pockets were discarded. A detailed list of the structures with the full annotations are available in Additional files 2, 3, 4 and 5: Information for serine (117 structures—Table S1), cysteine (95 structures—Table S2), aspartic (49 structures—Table S3) and threonine proteases (49 structures—Table S4).

#### Classification of structures based on MEROPS family information

Using the MEROPS database classification, the proteases were split into four categories: serine, cysteine, aspartic and threonine proteases. For some protease families, we found representative structures of subfamilies/classes. This is the case for serine proteases in MEROPS family S01 (serine endopeptidases) and subfamily S1A, which contains a set of structurally characterized homologues such as thrombin and trypsin 1. In total, we selected eight representative PDB structures for this subfamily. They were chosen to have one structure per class with the longest peptide bound. This set of serine protease structures was split into those bound to peptides composed exclusively of natural amino acids, and those containing non-natural amino acids (NNAAs). From the latter set, we selected substrates containing a maximum of only one modified amino acid in any position of the sequence, to preserve as much as possible the true conformation of the peptide.

### Modelling workflow

#### Modelling of full peptide substrates composed of natural amino acids

Since we only want to analyse protease-peptide complexes which could occur in nature, but we do not want to lose information from the complex structures with peptide substrates containing NNAAs, or with missing amino acids in the P4-P4ʹ positions, we used a modelling approach as outlined below.

First, the MEROPS database (release 12.1) was accessed to retrieve information about known peptide substrates. According to the selected substrate sequences, a position-by-position alignment was run to map which natural amino acid should replace the NNAA originally found in the structure. Second, the mapped substrates were filtered based on the similarity of the NNAA to the amino acid to be replaced, according to a metric calculated using the RDKit package (https://www.rdkit.org/). Here, Morgan fingerprint representations of the NNAA and the natural amino acid were compared using the Tanimoto coefficient [49]. The closer the values to 1.0, the more similar the side chains. The Rosetta fixbb tool [50] was applied to predict the new side chain’s conformation. The mutation protocol uses the Dunbrack rotamer libraries to select the most probable rotamer of the new amino acid [51]. After that, the system was relaxed using Rosetta with only side chains treated as flexible.

The next task was to model any missing residues at the P4-P4ʹ positions. The same substrates chosen from the MEROPS database were used as references to identify which amino acids should be included in the missing flanking region. The Modeller software [52] was employed to perform the reconstruction, using the bound peptide as template to model the missing part. The best model was subjected to a final relaxation of side chains with Rosetta. After the modelling, a list of proteases bound to 8-mer peptides that are part of known protein substrates was generated. A graphical example of the modelling and reconstruction is shown in Additional file 1: Fig. S4.

### Binding recognition analysis of some protease-peptide complexes

For this analysis, we selected a small subset of the modelled protease-peptide complexes to run simulations and capture structural observables that might correlate with enzyme binding recognition, as characterized by information-entropies derived from experimental cleavage data.

#### Selection of protease structures and entropy calculation

From the list of proteases available, structures were selected from each class for the simulations. The classes were chosen based on the availability of experimental cleavage data, which was used to create an experimental measure (from an information perspective) of the specificity of each position in the peptide template. The cleavage entropy of each position is a measure used to rank-order proteases by specificity, and is defined as [8]:

$$\mathrm{H}\left(\mathrm{j}\right)=-{\sum }_{\mathrm{i}=1}^{20}{\mathrm{n}}_{\mathrm{ij}}{\mathrm{log}}_{20}\left({\mathrm{n}}_{\mathrm{ij}}\right),$$

where *n*_*ij*_ is the occurrence of amino acid i at position j of the S4-S4ʹ binding region, divided by the total number of protease substrates. According to the formula, the single position entropy, *H(j),* ranges from 0 to 1, where 0 means absolute prevalence of a certain amino acid and 1 means equal usage of all amino acids. Using the calculated *H(j),* we obtained the total cleavage per subfamily/class by:

$${H}_{cleavage}={\sum }_{j=1}^{8}H\left(j\right),$$

where *H*_*cleavage*_ is the total cleavage entropy, which ranges between 0 and 8, and represents the sum of the eight positions.

#### Modelling of random peptide libraries

Based on each protease-peptide complex selected, we modelled two independent random libraries of 480 peptides, using the initial bound peptide conformation as template. The libraries were designed randomly with a uniform distribution of the amino acids at each position in the P4-P4ʹ region. Total coverage would require 8^20^ peptides, but for this analysis we limited the number of computational calculations to provide a fairly broad exploration of peptide binding. The idea was to observe the influence of each amino acid at each position. The peptides were modelled by iterative single substitutions of each amino acid in the template by a new amino acid from the peptide library, using the Rosetta fixbb protocol. After each mutation, a relaxation phase was run with a posterior refinement of the complex using the FlexPepDock protocol from Rosetta [53].

#### Dynamic analysis

For each optimized protease-peptide model from the random libraries, a dynamic analysis was run to sample not only the side chain conformations, but also the backbone of both the protein and the peptide. For this purpose, the Backrub method from Rosetta was used [38]. This employs a Monte Carlo mover that allows dihedral rotations and translations of the structure using a Metropolis criterion based on bond-angle penalties from reference force fields. The simulations were run for 5000 Monte Carlo steps, with a kT factor of 1.2 to allow more flexibility of the system without losing stability [54]. A total of 500 frames per complex were extracted. The Monte Carlo simulations were used to sample the systems with computational efficiency. They enable the exploration of the conformational space around the complex minimum without requiring massive computational resources, as in the case of molecular dynamics or more exhaustive approaches.

#### Calculation of structural observables and comparisons

From the frames obtained, a set of observables were calculated per position in the peptide. Specifically, we calculated the number of potential hydrogen bonds made by the main and side chain atoms, the number of non-bonded interactions made by the main and side chain atoms, the relative accessible surface area (ASA) and a single interaction energy associated with each amino acid. The hydrogen bonds and non-bonded contacts were calculated using HBPLUS [55]. The accessible surface area was calculated with DSSP [56] using BioPython functionalities [57], and the interaction energies were calculated using the Rosetta scoring function [58].

**Fig. 7:**
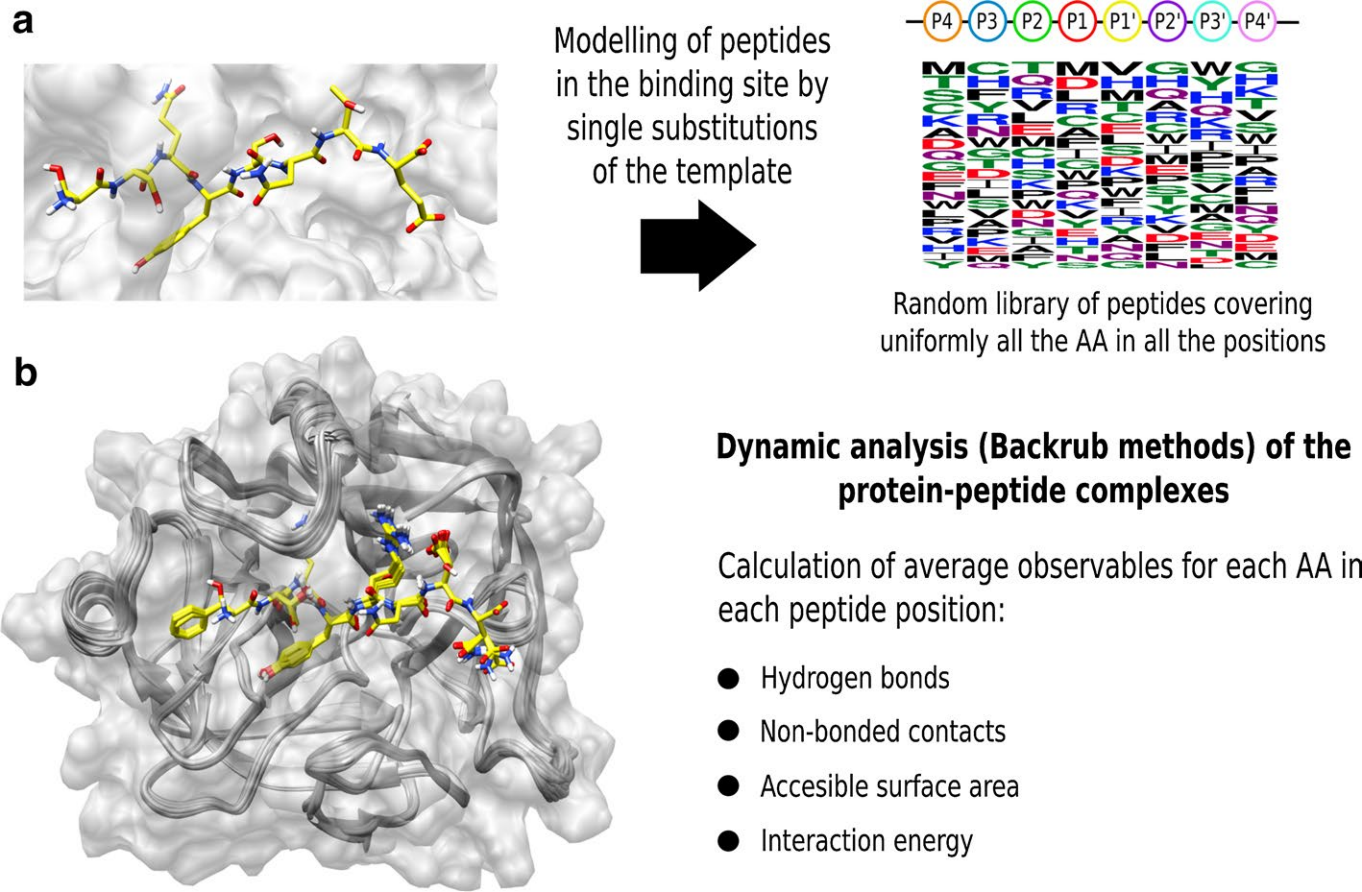
Summary of the modelling and simulation approaches to obtain information on binding recognition at each peptide position for all the amino acids-AA. **a** Generation of a random library of peptides that was modelled by single substitutions on the peptide template. **b** Dynamic analysis and calculation of different interaction observables using the modelled complexes

We calculated averages of the observables per amino acid in each position of the peptide substrate. At the *j*^*th*^ position for amino acid type *i*, the average value of observable *O* is defined as

$${O}_{ij}=\frac{1}{{\text{N}}_{\text{f}}}\sum_{\mathrm{\alpha }}\sum_{f}{o}_{ij}^{\alpha {\text{f}}},$$

where *o* is the observable, *f* is the frame number, *N*_*f*_ is the total number of frames and α indexes the simulation run (having one simulation for each binding-peptide from the dataset). Then, to compare the values with the previous calculated information entropies, we averaged the observed values for all the amino acids at each position in the P4-P4ʹ region. The comparison was based on observing the averages of the structural descriptors, and then checked to see which are able to describe better the experimental entropies reported for each binding pocket. A summary of the modelling/dynamic protocols used in this section is shown in Fig. 7.

## Supplementary Information

**Additional file 1:** Supplementary figures and tables.

**Additional file 2:** List of annotated serine proteases.

**Additional file 3:** List of annotated cysteine proteases.

**Additional file 4:** List of annotated aspartic proteases.

**Additional file 5:** List of annotated threonine proteases.

## Data Availability

The code and the protease-annotated data is available as a GitHub repository from the following link: https://github.com/rochoa85/Modelling-Protease-Substrates. A second repository with the scripts required to do the annotation process is available at: https://github.com/magnitov/protease_annotation_pipeline. Any questions related to the implementation can be directed to the registered author’s email account.

## References

[1] Marnett AB, Craik CS (2005). Papa’s got a brand new tag: advances in identification of proteases and their substrates. Trends Biotechnol.

[2] Puente XS, Sanchez LM, Overall CM, Lopez-Otin C (2003). Human and mouse proteases: a comparative genomic approach. Nat Rev Genet.

[3] Gurumallesh P, Alagu K, Ramakrishnan B, Muthusamy S (2019). A systematic reconsideration on proteases. Int J Biol Macromol.

[4] Schechter I, Berger A (1967). On the size of the active site in proteases. I Papain Biochem Biophys Res Commun.

[5] Hedstrom L (2002). Serine protease mechanism and specificity. Chem Rev.

[6] Martin CE, List K (2019). Cell surface–anchored serine proteases in cancer progression and metastasis. Cancer Metastasis Rev.

[7] Enoksson M, Li J, Ivancic MM, Timmer JC, Wildfang E, Eroshkin A, Salvesen GS, Tao WA (2007). Identification of proteolytic cleavage sites by quantitative proteomics. J Proteome Res.

[8] Fuchs JE, von Grafenstein S, Huber RG, Margreiter MA, Spitzer GM, Wallnoefer HG, Liedl KR (2013). Cleavage entropy as quantitative measure of protease specificity. PLOS Comput Biol.

[9] Rawlings ND, Barrett AJ, Thomas PD, Huang X, Bateman A, Finn RD (2018). The MEROPS database of proteolytic enzymes, their substrates and inhibitors in 2017 and a comparison with peptidases in the PANTHER database. Nucleic Acids Res.

[10] Li Q, Yi L, Marek P, Iverson BL (2013). Commercial proteases: Present and future. FEBS Lett.

[11] Barkan DT, Hostetter DR, Mahrus S, Pieper U, Wells JA, Craik CS, Sali A (2010). Prediction of protease substrates using sequence and structure features. Bioinformatics.

[12] Song J, Tan H, Perry AJ, Akutsu T, Webb GI, Whisstock JC, Pike RN (2012). PROSPER: an integrated feature-based tool for predicting protease substrate cleavage sites. PLoS ONE.

[13] Chen CT, Yang EW, Hsu HJ, Sun YK, Hsu WL, Yang AS (2008). Protease substrate site predictors derived from machine learning on multilevel substrate phage display data. Bioinformatics.

[14] Garay-Malpartida HM, Occhiucci JM, Alves J, Belizario JE (2005). CaSPredictor: a new computer-based tool for caspase substrate prediction. Bioinformatics.

[15] Lohmüller T, Wenzler D, Hagemann S, Kieß W, Peters C, Dandekar T, Reinheckel T (2003). Toward computer-based cleavage site prediction of cysteine endopeptidases. Biol Chem.

[16] Singh O, Su EC-Y (2016). Prediction of hiv-1 protease cleavage site using a combination of sequence, structural, and physicochemical features. BMC Bioinformatics.

[17] Li F, Wang Y, Li C, Marquez-Lago TT, Leier A, Rawlings ND, Haffari G, Revote J, Akutsu T, Chou K-C, Purcell AW, Pike RN, Webb GI, Ian Smith A, Lithgow T, Daly RJ, Whisstock JC, Song J (2018). Twenty years of bioinformatics research for protease-specific substrate and cleavage site prediction: a comprehensive revisit and benchmarking of existing methods. Brief Bioinform.

[18] Song J, Li F, Leier A, Marquez-Lago TT, Akutsu T, Haffari G, Chou KC, Webb GI, Pike RN (2018). PROSPERous: high-throughput prediction of substrate cleavage sites for 90 proteases with improved accuracy. Bioinformatics.

[19] Rognvaldsson T, You L, Garwicz D (2015). State of the art prediction of HIV-1 protease cleavage sites. Bioinformatics.

[20] Artimo P, Jonnalagedda M, Arnold K, Baratin D, Csardi G, De Castro E, Duvaud S, Flegel V, Fortier A, Gasteiger E, Grosdidier A, Hernandez C, Ioannidis V, Kuznetsov D, Liechti R, Moretti S, Mostaguir K, Redaschi N, Rossier G, Xenarios I, Stockinger H (2012). ExPASy: SIB bioinformatics resource portal. Nucleic Acids Res.

[21] Vinothkumar KR, Strisovsky K, Andreeva A, Christova Y, Verhelst S, Freeman M (2010). The structural basis for catalysis and substrate specificity of a rhomboid protease. EMBO J.

[22] Zhang C, Srinivasan Y, Arlow DH, Fung JJ, Palmer D, Zheng Y, Green HF, Pandey A, Dror RO, Shaw DE, Weis WI, Coughlin SR, Kobilka BK (2012). High-resolution crystal structure of human protease-activated receptor 1. Nature.

[23] Leung D, Abbenante G, Fairlie DP (2000). Protease inhibitors: Current status and future prospects. J Med Chem.

[24] Verma S, Dixit R, Pandey KC (2016). Cysteine proteases: modes of activation and future prospects as pharmacological targets. Front Pharmacol.

[25] Drag M, Salvesen GS (2010). Emerging principles in protease-based drug discovery. Nat Rev Drug Discov.

[26] Fear G, Komarnytsky S, Raskin I (2007). Protease inhibitors and their peptidomimetic derivatives as potential drugs. Pharmacol Therapeut.

[27] Radisky ES, Lee JM, Lu CJK, Koshland DE (2006). Insights into the serine protease mechanism from atomic resolution structures of trypsin reaction intermediates. Proc Natl Acad Sci USA.

[28] Kumar N, Sood D, van der Spek PJ, Sharma HS, Chandra R (2020). Molecular binding mechanism and pharmacology comparative analysis of noscapine for repurposing against SARS-CoV-2 protease. J Proteome Res.

[29] Barman A, Schürer S, Prabhakar R (2011). Computational modeling of substrate specificity and catalysis of the β-secretase (BACE1) enzyme. Biochemistry.

[30] Perez MAS, Fernandes PA, Ramos MJ (2010). Substrate recognition in HIV-1 protease: a computational study. J Phys Chem B.

[31] Pethe MA, Rubenstein AB, Khare SD (2019). Data-driven supervised learning of a viral protease specificity landscape from deep sequencing and molecular simulations. Proc Natl Acad Sci.

[32] Ribeiro AJM, Holliday GL, Furnham N, Tyzack JD, Ferris K, Thornton JM (2018). Mechanism and Catalytic Site Atlas (M-CSA): a database of enzyme reaction mechanisms and active sites. Nucleic Acids Res.

[33] Guerin ME, Stirnemann G, Giganti D (2018). Conformational entropy of a single peptide controlled under force governs protease recognition and catalysis. Proc. Natl. Acad. Sci..

[34] Fuchs JE, von Grafenstein S, Huber RG, Wallnoefer HG, Liedl KR (2014). Specificity of a protein-protein interface: local dynamics direct substrate recognition of effector caspases. Proteins Struct Funct Bioinf.

[35] Van Der Kamp MW, Mulholland AJ (2013). Combined quantum mechanics/molecular mechanics (QM/MM) methods in computational enzymology. Biochemistry.

[36] Rubenstein AB, Pethe MA, Khare SD (2017). MFPred: Rapid and accurate prediction of protein-peptide recognition multispecificity using self-consistent mean field theory. PLOS Comput Biol.

[37] Berman HM, Westbrook J, Feng Z, Gilliland G, Bhat TN, Weissig H, Shindyalov IN, Bourne PE (2000). The protein data bank. Nucleic Acids Res.

[38] Smith CA, Kortemme T (2008). Backrub-like backbone simulation recapitulates natural protein conformational variability and improves mutant side-chain prediction. J Mol Biol.

[39] Barlow KA, Conchúir O, S., Thompson, S., Suresh, P., Lucas, J.E., Heinonen, M., Kortemme, T. (2018). Flex ddG: Rosetta ensemble-based estimation of changes in protein–protein binding affinity upon mutation. J. Phys. Chem. B.

[40] Smith CA, Kortemme T (2011). Predicting the tolerated sequences for proteins and protein interfaces using rosetta-backrub flexible backbone design. PLoS ONE.

[41] Pethe MA, Rubenstein AB, Khare SD (2017). Large-scale structure-based prediction and identification of novel protease substrates using computational protein design. J Mol Biol.

[42] Westbrook JD, Shao C, Feng Z, Zhuravleva M, Velankar S, Young J (2015). The chemical component dictionary: complete descriptions of constituent molecules in experimentally determined 3D macromolecules in the Protein Data Bank. Bioinformatics.

[43] Laskowski RA, Jablonska J, Pravda L, Varekova RS, Thornton JM (2018). PDBsum: structural summaries of PDB entries. Protein Sci.

[44] Bairoch, A.: The Universal Protein Resource (UniProt). Nucleic Acids Res. 33(Database issue), 154–159 (2004) 10.1093/nar/gki070 PMC540024 15608167

[45] Finn RD, Tate J, Mistry J, Coggill PC, Sammut SJ, Hotz HR, Ceric G, Forslund K, Eddy SR, Sonnhammer ELL, Bateman A (2008). The Pfam protein families database. Nucleic Acids Res.

[46] Greene LH, Lewis TE, Addou S, Cuff A, Dallman T, Dibley M, Redfern O, Pearl F, Nambudiry R, Reid A, Sillitoe I, Yeats C, Thornton JM, Orengo CA (2007). The CATH domain structure database: new protocols and classification levels give a more comprehensive resource for exploring evolution. Nucleic Acids Res.

[47] Chenna R, Sugawara H, Koike T, Lopez R, Gibson TJ, Higgins DG, Thompson JD (2003). Multiple sequence alignment with the Clustal series of programs. Nucleic Acids Res.

[48] Cuesta SA, Mora JR, Zambrano CH, Torres FJ, Rincón L (2020). Comparative study of the nucleophilic attack step in the proteases catalytic activity: A theoretical study. Mol Phys.

[49] Bajusz D, Rácz A, Héberger K (2015). Why is Tanimoto index an appropriate choice for fingerprint-based similarity calculations?. J Cheminformatics.

[50] Loffler P, Schmitz S, Hupfeld E, Sterner R, Merkl R, Hughes M (2017). Rosetta:MSF: a modular framework for multi-state computational protein design. PLOS Comput Biol.

[51] Dunbrack RL, Karplus M (1994). Conformational analysis of the backbone-dependent rotamer preferences of protein sidechains. Nat Struct Mol Biol.

[52] Marti-Renom MA, Stuart AC, Sanchez R, Melo F, Sali A (2000). Comparative protein structure modeling of genes and genomes. Annu Rev Biophys Biomol Struct.

[53] Raveh B, London N, Schueler-Furman O (2010). Sub-angstrom modeling of complexes between flexible peptides and globular proteins. Proteins Struct Funct Bioinf.

[54] Loshbaugh AL, Kortemme T (2020). Comparison of Rosetta flexible-backbone computational protein design methods on binding interactions. Proteins.

[55] McDonald IK, Thornton JM (1994). Satisfying hydrogen bonding potential in proteins. J Mol Biol.

[56] Kabsch W, Sander C (1983). Dictionary of protein secondary structure: Pattern recognition of hydrogen-bonded and geometrical features. Biopolymers.

[57] Cock PJA, Antao T, Chang JT, Chapman BA, Cox CJ, Dalke A, Friedberg I, Hamelryck T, Kauff F, Wilczynski B, De Hoon MJL (2009). Biopython: Freely available Python tools for computational molecular biology and bioinformatics. Bioinformatics.

[58] Alford RF, Leaver-Fay A, Jeliazkov JR, O’Meara MJ, DiMaio FP, Park H, Shapovalov MV, Renfrew PD, Mulligan VK, Kappel K, Labonte JW, Pacella MS, Bonneau R, Bradley P, Dunbrack RL, Das R, Baker D, Kuhlman B, Kortemme T, Gray JJ (2017). The Rosetta all-atom energy function for macromolecular modeling and design. J Chem Theory Comput.

[59] Zoll S, Stanchev S, Began J, Skerle J, Lepsık M, Peclinovska L, Majer P, Strisovsky K (2014). Substrate binding and specificity of rhomboid intramembrane protease revealed by substrate–peptide complex structures. EMBO J.

[60] Liang L, Liu S, Yang J, Meng Z, Lei L, Zhang K (2011). Comparison of homology models and crystal structures of cuticle-degrading proteases from nematophagous fungi: structural basis of nematicidal activity. FASEB J.

[61] Farinas ET, Bulter T, Arnold FH (2001). Directed enzyme evolution.. Curr Opin Biotech.

[62] Nobeli I, Favia AD, Thornton JM (2009). Protein promiscuity and its implications for biotechnology. Nat Biotechnol.

[63] Hult K, Berglund P (2007). Enzyme promiscuity: mechanism and applications. Trends Biotechnol.

[64] Lutz S (2010). Beyond directed evolution-semi-rational protein engineering and design. Curr Opin Biotech.

[65] Ma S, Devi-Kesavan LS, Gao J (2007). Molecular dynamics simulations of the catalytic pathway of a cysteine protease: a combined QM/MM study of human cathepsin K. J Am Chem Soc.

[66] Lima MCP, Seabra GM (2016). Reaction mechanism of the dengue virus serine protease: a QM/MM study. Phys Chem Chem Phys.

[67] Renfrew PD, Choi EJ, Bonneau R, Kuhlman B (2012). Incorporation of noncanonical amino acids into rosetta and use in computational protein-peptide interface design. PLoS ONE.

