# Supplementary material for: An automated protocol for modelling peptide substrates to proteases
Source: BMC Bioinformatics. 2020 Dec 29;21:586. doi: 10.1186/s12859-020-03931-6 (PMC7771086; doi:10.1186/s12859-020-03931-6)
Supplement: Supplementary file 1 — Additional file 1: Supplementary figures and tables. [file 12859_2020_3931_MOESM1_ESM.docx]

**Supplementary Information**

**An automated protocol for modelling peptide substrates to proteases**

Rodrigo Ochoa^1,2^, Mikhail Magnitov^2,#^, Roman A. Laskowski^2^, Pilar Cossio^1,3^, Janet M. Thornton^2^

^1^Biophysics of Tropical Diseases, Max Planck Tandem Group, University of Antioquia, 050010 Medellín, Colombia.

^2^European Molecular Biology Laboratory, European Bioinformatics Institute (EMBL-EBI), Wellcome Trust Genome Campus, Hinxton, Cambridge CB10 1SD, United Kingdom

^3^Department of Theoretical Biophysics, Max Planck Institute of Biophysics, 60438 Frankfurt am Main, Germany.

^#^Current address: Department of Biological and Medical Physics, Moscow Institute of Physics and Technology (National Research University), Dolgoprudny 141701, Russia.

**Supplementary Figures**

**
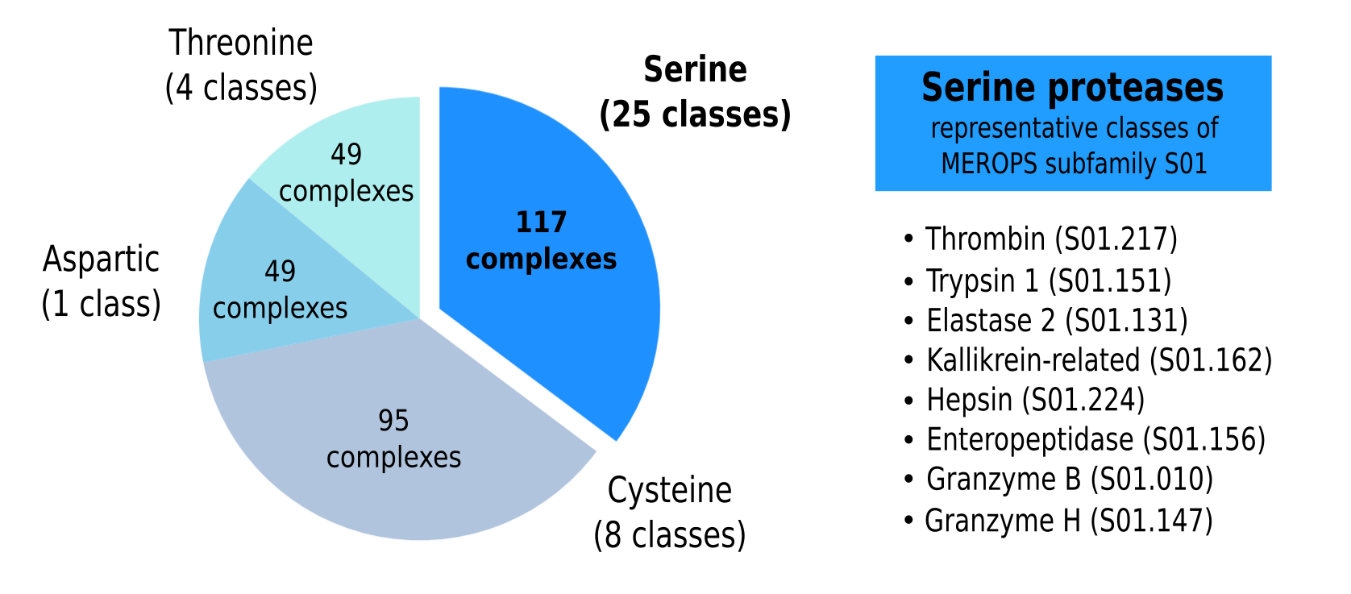
**

**Fig. S1**. Distribution of the structures detected during the annotation phase. The serine proteases are the most diverse group with 117 crystal structures covering 25 classes from MEROPS database, where the subfamily S01 of serine endopeptidases is the most representative.


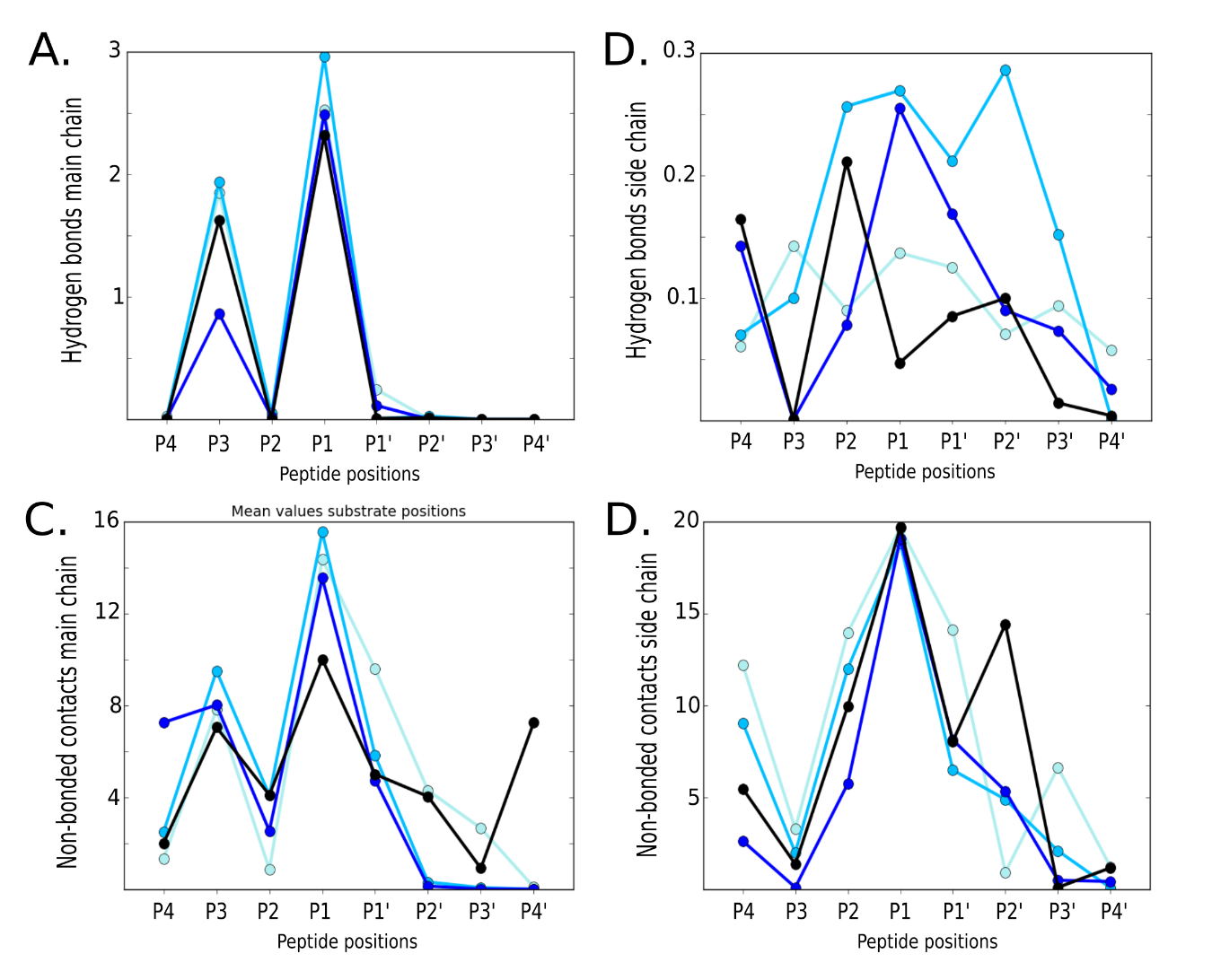


**Fig. S2**. Mean values of structural observables at each position of the peptides modelled for structures 4dt7 (MEROPS id S01.217) in turquoise, 1iau (MEROPS id S01.010) in sky blue, 1tps (MEROPS id S01.151) in blue, and 1ppg (MEROPS id S01.131) in black. The observables included are the number of hydrogen bonds between the protein and the peptide’s main chain (A) and side chain atoms, and the number of non-bonded contacts between the protein and the peptide’s main chain (C) and side chain atoms.


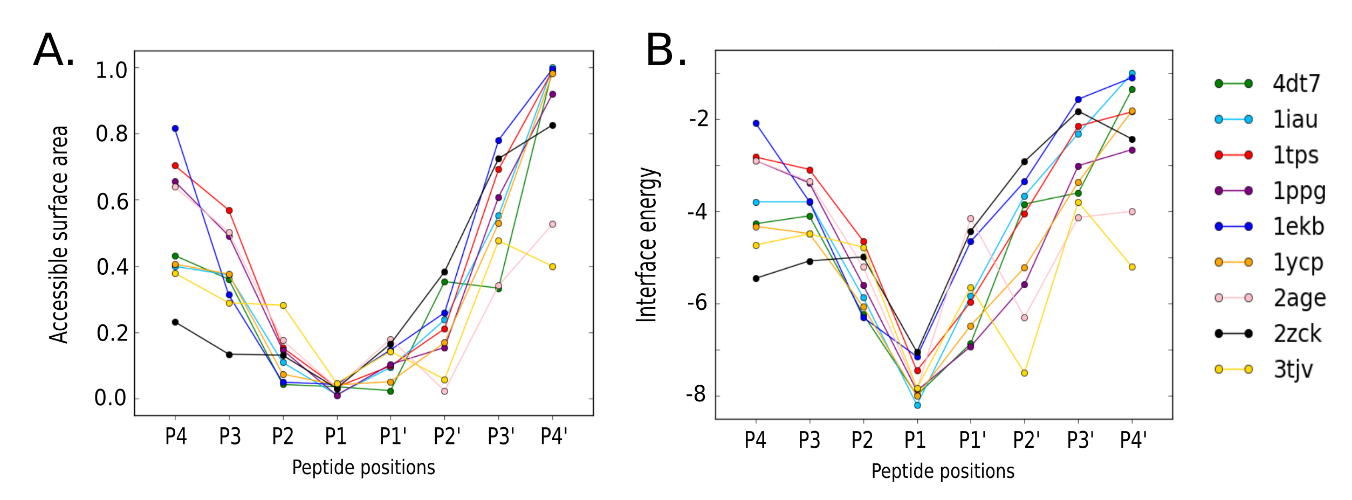


**Fig. S3**. Mean values of structural observables at each position of the peptides modelled for structures 4dt7 (MEROPS id S01.217) in green, 1iau (MEROPS id S01.010) in cyan, 1tps (MEROPS id S01.151) in red, 1ppg (MEROPS id S01.131) in purple, 1ekb (MEROPS id S01.156) in blue, 1ycp (MEROPS id S01.217) in gold, 2age (MEROPS id S01.151) in pink, 2zck (MEROPS id S01.162) in black, and 3tjv (MEROPS id S01.147) in yellow. The observables included are the interaction energy calculated using the Rosetta scoring function (A), and the relative accessible surface area -ASA (B).


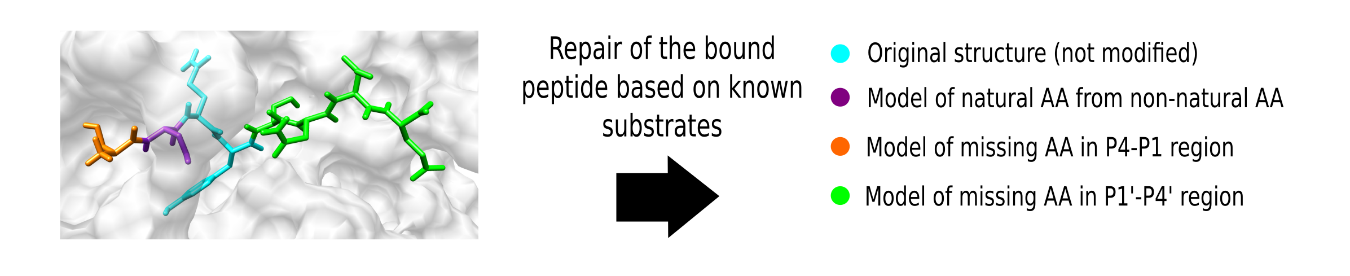


**Fig. S4**. Schematic of the substrate modelling, including the original crystal structure (cyan), the modelled natural AA instead of the non-natural AA present (purple), and the model of missing amino acids in the P4-P1 region (orange) and P1’-P4’ region (green).

**Supplementary Tables**

**Table S1.** List of annotated serine proteases structure from PDB, including the id, ligand sequence, ligand size, ligand chain, ligand residue numbers, protein chains, protein residues, EC number, CATH domains, Pfam domains, UniProt id, MEROPS id, catalytic residues, catalytic roles, structure index, truncated ligand sequence, truncated ligand numbering and. truncated pocket names. **[The table is available as an external xlsx file].**

**Table S2.** List of annotated cysteine proteases structure from PDB, including the id, ligand sequence, ligand size, ligand chain, ligand residue numbers, protein chains, protein residues, EC number, CATH domains, Pfam domains, UniProt id, MEROPS id, catalytic residues, catalytic roles, structure index, truncated ligand sequence, truncated ligand numbering and. truncated pocket names. **[The table is available as an external xlsx file].**

**Table S3.** List of annotated aspartic proteases structure from PDB, including the id, ligand sequence, ligand size, ligand chain, ligand residue numbers, protein chains, protein residues, EC number, CATH domains, Pfam domains, UniProt id, MEROPS id, catalytic residues, catalytic roles, structure index, truncated ligand sequence, truncated ligand numbering and. truncated pocket names. **[The table is available as an external xlsx file].**

**Table S4.** List of annotated threonine proteases structure from PDB, including the id, ligand sequence, ligand size, ligand chain, ligand residue numbers, protein chains, protein residues, EC number, CATH domains, Pfam domains, UniProt id, MEROPS id, catalytic residues, catalytic roles, structure index, truncated ligand sequence, truncated ligand numbering and. truncated pocket names. **[The table is available as an external xlsx file].**

**Table S5.** List of serine protease structures with peptides modelled. The MEROPS id, substrate UniProt id and the peptide sequences after modelling are provided.

| **PDB** | **MEROPS** | **Template** | **Model** | **Substrate UniProt id** |
| --- | --- | --- | --- | --- |
| 1dx5 | S01.217 | -EGR---- | LEGRYFSG | P31431 |
| 1ekb | S01.156 | -DDK---- | DDDKIVGG | P35030 |
| 1iau | S01.010 | IEPD---- | IEPDTDAP | P50502 |
| 1iau | S01.010 | IEPE---- | IEPESGKS | Q8WXA9 |
| 1nrs | S01.217 | LDPR---- | LDPRSFLL | P25116 |
| 1ppg | S01.131 | AAPA---- | AAPAAAPP | P16403 |
| 1tmt | S01.217 | -EPR---- | IEPRSFSQ | P00451 |
| 1tmt | S01.217 | -DPR---- | VDPRLIDG | P04070 |
| 1tmt | S01.217 | -SPR---- | LSPRTFHP | P12259 |
| 1tps | S01.151 | -LTREL-- | FLTRELAE | P23396 |
| 1tps | S01.151 | -PTREL-- | APTRELAQ | P60842 |
| 1ycp | S01.217 | -GVRGP-- | GGVRGPRV | P02671 |
| 1z8g | S01.224 | KQLR---- | KQLRVVNG | P14210 |
| 2age | S01.151 | AAPR---- | AAPRELGL | Q9BTD8 |
| 2age | S01.151 | AAPR---- | AAPRERTT | Q13895 |
| 2age | S01.151 | AAPR---- | AAPRTFLR | Q9UDY2 |
| 2agg | S01.151 | AAPK---- | AAPKVRFM | P61088 |
| 2agg | S01.151 | AAPK---- | AAPKRVEI | Q9UHD8 |
| 2agi | S01.151 | -LLK---- | ALLKNYGL | P01242-3 |
| 2agi | S01.151 | -LLR---- | LLLRRKFF | Q9Y490 |
| 2agi | S01.151 | -LLR---- | ELLRSAQP | Q9Y490 |
| 2hgt | S01.217 | -NPR---- | FNPRTFGS | P00734 |
| 2hgt | S01.217 | -KPR---- | IKPRIVGG | P03951 |
| 2hgt | S01.217 | -RPR---- | LRPRFKII | P00749 |
| 2zck | S01.162 | -SQY---- | SSQYSNTE | P04279 |
| 3bf6 | S01.217 | -FPR---- | AFPRVKPA | Q12905 |
| 3hat | S01.217 | QGVR---- | QGVRGYPT | Q8NBS9 |
| 3qdz | S01.217 | PAPR---- | PAPRGYPG | Q96RI0 |
| 3vxe | S01.217 | -VPR---- | VVPRGVNL | P00488 |
| 3vxe | S01.217 | -SPR---- | LSPRGVHI | P17936 |
| 3vxe | S01.217 | -HPR---- | AHPRIISA | P24593 |
| 4boh | S01.217 | -KPR---- | IKPRIVGG | P03951 |
| 4boh | S01.217 | -KPR---- | LKPRVGKA | P42285 |
| 4dt7 | S01.217 | VDPRL--- | VDPRLIDG | P04070 |

**Table S6.** List of cysteine protease structures with peptides modelled. The MEROPS id, substrate UniProt id and the peptide sequences after modelling are provided.

| **PDB** | **MEROPS** | **Template** | **Model** | **Substrate UniProt id** |
| --- | --- | --- | --- | --- |
| 4qu9 | C14.003 | DEVD---- | DEVDGVDE | P09874 |
| 4qu9 | C14.003 | DEVD---- | DEVDSKRL | Q01082 |
| 4qu9 | C14.003 | DEVD---- | DEVDNKVK | P78527 |
| 4jr2 | C14.004 | DEVD---- | DEVDGLGV | P46940 |
| 2j31 | C14.003 | DEVD---- | DEVDGMAG | P35251 |
| 2j31 | C14.003 | DEVD---- | DEVDSLMC | Q16513 |
| 2j31 | C14.003 | DEVD---- | DEVDKMCH | Q04759 |
| 2j30 | C14.003 | DEVD---- | DEVDGLGV | P46940 |
| 2j30 | C14.003 | DEVD---- | DEVDQDGN | Q9Y6H5 |
| 2j30 | C14.003 | DEVD---- | DEVDGEEQ | Q9UEE9 |
| 2j33 | C14.003 | DEVD---- | DEVDSLKE | Q15056 |
| 3d6m | C14.001 | CVAD---- | CVADALGA | Q96JH7 |
| 4dco | C14.003 | DEVD---- | DEVDGMAG | P35251 |
| 4hva | C14.005 | VEID---- | VEIDNGKQ | P02545 |
| 1f1j | C14.004 | DEVE---- | DEVELARI | O15355 |
| 2h5j | C14.003 | DMQD---- | DMQDNSGT | Q05655 |
| 2cnn | C14.003 | IETD---- | IETDKATI | P10515 |
| 2c2z | C14.009 | LETD---- | LETDGGGP | Q8WUI4 |
| 4jr0 | C14.003 | DEVD---- | DEVDGVDE | P09874 |
| 4dcp | C14.003 | DEVD---- | DEVDSLKE | Q15056 |
| 2ql7 | C14.004 | IEPD---- | IEPDTPGR | Q15149 |
| 3h11 | C14.009 | IETD---- | IETDSGVD | P42574 |
| 4jr1 | C14.004 | DEVE---- | DEVERVIT | P62269 |

**Table S7.** Average Spearman correlation between the structural observables and the entropy calculated from the experimental cleavage data for the four serine protease subfamily classes 4dt7 (MEROPS id S01.217), 1iau (MEROPS id S01.010), 1tps (MEROPS id S01.151) and 1ppg (MEROPS id S01.131).

| **Observable** | **Average Spearman correlation** |
| --- | --- |
| Relative ASA | 0.3 |
| Interface energy | 0.22 |
| Hydrogen bonds main chain | -0.45 |
| Hydrogen bonds side chain | -0.17 |
| Non-bonded contacts main chain | -0.25 |
| Non-bonded contacts side chain | -0.35 |
